# Supplementary material for: Role and mechanism of NCAPD3 in promoting malignant behaviors in gastric cancer
Source: Front Pharmacol. 2024 Apr 22;15:1341039. doi: 10.3389/fphar.2024.1341039 (PMC11070777; doi:10.3389/fphar.2024.1341039)
Supplement: Supplementary file 11 [file DataSheet2.ZIP › GSEA/immunologic/my_analysis.Gsea.1599462003817/GSE18791_CTRL_VS_NEWCASTLE_VIRUS_DC_2H_UP.html]

Details for gene set GSE18791\_CTRL\_VS\_NEWCASTLE\_VIRUS\_DC\_2H\_UP[GSEA]

|  || Dataset | filtered\_dataset.sample\_info.cls#WT\_versus\_NCAPD3\_MUT |
| Phenotype | sample\_info.cls#WT\_versus\_NCAPD3\_MUT |
| Upregulated in class | WT |
| GeneSet | GSE18791\_CTRL\_VS\_NEWCASTLE\_VIRUS\_DC\_2H\_UP |
| Enrichment Score (ES) | 0.41237494 |
| Normalized Enrichment Score (NES) | 2.0538256 |
| Nominal p-value | 0.001610306 |
| FDR q-value | 0.8063549 |
| FWER p-Value | 0.981 |
Table: GSEA Results Summary

  

Fig 1: Enrichment plot: GSE18791\_CTRL\_VS\_NEWCASTLE\_VIRUS\_DC\_2H\_UP      
 Profile of the Running ES Score & Positions of GeneSet Members on the Rank Ordered List

  

| SYMBOL | TITLE | RANK IN GENE LIST | RANK METRIC SCORE | RUNNING ES | CORE ENRICHMENT || 1 | 222236 | NAPEPLD | 36 | 0.977 | 0.0281 | Yes |
| 2 | 55781 | RIOK2 | 135 | 0.763 | -0.0005 | Yes |
| 3 | 11162 | NUDT6 | 162 | 0.716 | 0.0204 | Yes |
| 4 | 57325 | CSRP2BP | 176 | 0.698 | 0.0498 | Yes |
| 5 | 5189 | PEX1 | 200 | 0.676 | 0.0707 | Yes |
| 6 | 54801 | HAUS6 | 219 | 0.660 | 0.0943 | Yes |
| 7 | 55028 | C17orf80 | 222 | 0.659 | 0.1294 | Yes |
| 8 | 146050 | ZSCAN29 | 226 | 0.654 | 0.1636 | Yes |
| 9 | 51250 | C6orf203 | 230 | 0.650 | 0.1975 | Yes |
| 10 | 221504 | ZBTB9 | 237 | 0.647 | 0.2291 | Yes |
| 11 | 25974 | MMACHC | 265 | 0.621 | 0.2441 | Yes |
| 12 | 25973 | PARS2 | 266 | 0.620 | 0.2785 | Yes |
| 13 | 116143 | WDR92 | 267 | 0.619 | 0.3129 | Yes |
| 14 | 84078 | KBTBD7 | 295 | 0.594 | 0.3263 | Yes |
| 15 | 9724 | UTP14C | 301 | 0.591 | 0.3555 | Yes |
| 16 | 8125 | ANP32A | 326 | 0.578 | 0.3702 | Yes |
| 17 | 85315 | PAQR8 | 351 | 0.567 | 0.3843 | Yes |
| 18 | 5001 | ORC5 | 450 | 0.506 | 0.3413 | Yes |
| 19 | 10940 | POP1 | 497 | 0.474 | 0.3343 | Yes |
| 20 | 83448 | PUS7L | 520 | 0.462 | 0.3440 | Yes |
| 21 | 29090 | TIMM21 | 537 | 0.453 | 0.3576 | Yes |
| 22 | 2188 | FANCF | 545 | 0.449 | 0.3775 | Yes |
| 23 | 93134 | ZNF561 | 590 | 0.431 | 0.3695 | Yes |
| 24 | 55039 | TRMT12 | 599 | 0.424 | 0.3872 | Yes |
| 25 | 55181 | SMG8 | 602 | 0.422 | 0.4092 | Yes |
| 26 | 643836 | ZFP62 | 639 | 0.405 | 0.4056 | Yes |
| 27 | 78996 | C7orf49 | 661 | 0.396 | 0.4124 | Yes |
| 28 | 5987 | TRIM27 | 695 | 0.380 | 0.4096 | No |
| 29 | 51000 | SLC35B3 | 740 | 0.355 | 0.3974 | No |
| 30 | 27300 | ZNF544 | 759 | 0.343 | 0.4034 | No |
| 31 | 6947 | TCN1 | 1135 | -0.503 | 0.1594 | No |
| 32 | 57533 | TBC1D14 | 1304 | -0.708 | 0.0769 | No |
Table: GSEA details [plain text format]

  

Fig 2: GSE18791\_CTRL\_VS\_NEWCASTLE\_VIRUS\_DC\_2H\_UP      
 Blue-Pink O' Gram in the Space of the Analyzed GeneSet

  

Fig 3: GSE18791\_CTRL\_VS\_NEWCASTLE\_VIRUS\_DC\_2H\_UP: Random ES distribution      
 Gene set null distribution of ES for **GSE18791\_CTRL\_VS\_NEWCASTLE\_VIRUS\_DC\_2H\_UP**
